# Effect of the Production of Dried Fruits and Juice from Chokeberry (*Aronia melanocarpa* L.) on the Content and Antioxidative Activity of Bioactive Compounds

**DOI:** 10.3390/molecules21081098

**Published:** 2016-08-22

**Authors:** Jan Oszmiański, Sabina Lachowicz

**Affiliations:** Department of Fruit and Vegetable Processing, Wroclaw University of Environmental and Life Science, 37, Chełmońskiego Street, Wroclaw 51-630, Poland; jan.oszmianski@up.wroc.pl

**Keywords:** *Aronia melanocarpa*, powders, juices, polyphenol compounds, antioxidative activity

## Abstract

The aim of this study was to evaluate the production of dried fruits and juices from chokeberry as potential sources of bioactive compounds with beneficial effects on human health. Dry powders and juices from chokeberry were analyzed for the contents of sugars with high-performance liquid chromatography coupled with an evaporative light scattering detector (HPLC-ELSD method), and the antioxidant capacity was analyzed by the FRAP (ferric-reducing ability of plasma) and ABTS (2,2′-azino-bis(3-ethylbenzothiazoline-6-sulphonic acid)) tests. Polyphenols were identified by high performance liquid chromatography (HPLC) coupled with a tandem mass spectrometer and a photodiode-array detector (LC-PDA-ESI-MS/MS), and their quantitative analysis was carried out by UPLC-MS/MS (using a Q/TOF detector and a PDA detector). A total of 27 polyphenolic compounds was identified in chokeberry products, including 7 anthocyanins, 11 flavonols, 5 phenolic acids, 3 flavan-3-ols and 1 flavanone. Three anthocyanin derivatives were reported for the first time from chokeberry fruit. A higher activity of the bioactive compounds was determined in dried fruit pomace and in juice obtained from crushed fruits than in those from the whole fruits. In addition, the pomace was found to be a better material for the production of dry powders, compared to chokeberry fruits.

## 1. Introduction

Black chokeberry has gained interest as one of the richest sources of bioactive compounds, displaying a high biological and nutritive value [1,2,3]. It is a good source of vitamins, β-carotene, dietary fiber, minerals, sugars and organic acids [4]. The most important group of chokeberry compounds is represented by polyphenolic compounds, including flavonoids (flavonols, anthocyanins, flavan-3-ols) and phenolic acids [5,6,7], with contents ranging from 3.73 in juice, through 7.85 in fruits to 10.58 g/100 g dry matter (dm) in pomace [5]. Chokeberry is especially rich in anthocyanins and proanthocyanidins. High contents of these compounds are hardly ever found in other fruits [8,9]. An exception is red grapes, the skin of which is rich in anthocyanins and seeds in proanthocyanidins [10].

Polyphenolic compounds display antioxidative properties, reduce blood levels of the low-density lipoprotein (LDL) cholesterol fraction and triglycerides [11,12] and minimize the risk of metabolic diseases (e.g., diabetes, obesity). Chokeberry is applied in the treatment of cardiovascular diseases, to reduce blood pressure and to lower the blood level of glucose owing to its high biological value [13,14]. In addition, it displays anti-inflammatory, gastroprotective, antidiabetic and hepatoprotective properties [15,16].

Black chokeberry fruits are characterized by a tart and bitter taste caused by a high content of polyphenols, particularly proanthocyanidin-condensing tannins, phenolic acids and bitter eriodictyol-glucuronide flavanone and, therefore, are rarely used for direct consumption [17]. The important and valuable products for industry are fruit juice and fruit powders. The usability of raw material for juice production results from its stability during harvest, transport, storage and pressing. Moreover, the tart-bitter taste of the juice is attenuated during processing, because the concentration of compounds responsible for the taste is reduced. Drying of the pomace is one of the most important elements during the production of dietary supplements (pills or capsules), functional foods, snacks or tea infusions [18,19,20,21,22]. The drying process affects the chemical composition of the raw material, the quality and contents of its bioactive compounds and its appearance [23,24,25,26]. A significant issue that needs to be considered during the drying process is minimizing the degradation of thermolabile compounds. Therefore, the best method recommended to manufacture such products is lyophilization [27]. Moreover, in the production of chokeberry fruit powders, the drying process is impaired by the presence of wax on the fruit skin. In addition, the necessity of evaporating large volumes of water poses difficulties in achieving the desired consistency of dried fruits due to the presence of sugars that affect hygroscopicity and viscosity values.

So far, research conducted on black chokeberry has concerned its products and extracts [28], juice concentrates [29] and powders obtained using different drying methods [1]. Considering the above, the objective of this study was to evaluate the feasibility of improving the production process of chokeberry powders with a high content of health-promoting compounds compared to difficult to dry whole berries and of producing chokeberry juice with a mild taste. The chemical composition (dry matter, sugars-fructose, sorbitol and glucose), the content of polyphenolic compounds (UPLC-PDA-MS/MS) and the antioxidative activity (FRAP, ABTS) were analyzed in juices produced by pressing the crushed chokeberry fruits (JCF) and the uncrushed chokeberry fruits (JUF), as well as in lyophilized and ground powders made of whole dried fruits (PDF) and from pomace obtained from whole (PPUF) and crushed fruits (PPCF).

## 2. Results and Discussion

### 2.1. Major Chemical Compounds

Table 1 summarizes the results of the determinations of dry matter and sugar contents in the analyzed products. The pressing of crushed and whole fruits affected the contents of both dry matter and sugars in juices. The content of dry matter and sugars in JUF was 16.87% and 87.31 g/100 g dm (dry matter), respectively, and was higher compared to JCF, i.e., 15.46% and 85.24 g/100 g dm, respectively. Kulling et al. [4] reported a similar content of dry matter (15.5%) in commercially-produced chokeberry juice (100%, pasteurized, not from concentrated juice). The content of total sugars in the PDF was 46.15 g/100 g dm, which was 1.6- and 1.8-times higher compared to the PPUF and PPCF. This indicates that the dried powders made of pomace were significantly poorer in sugars than the PDF. This is beneficial, as they exhibit lower hygroscopicity and lower viscosity and are easier to disintegrate.

The contents of individual sugars were found to differ among the analyzed products. In PDF, the contents of sorbitol, glucose and fructose were 21.08, 14.64 and 10.43 g/100 g dm. In the case of PPUF, the contents of sorbitol and glucose were at the same level, i.e., 12.55 g/100 g dm, whereas fructose content was lower by ca. 50%. In PPCF, the content of sorbitol was 13.33 g/100 g dm, that of fructose 6.64 g/100 g dm and that of glucose 5.08 g/100 g dm [4].

### 2.2. Identification of Phenolic Compounds in Black Chokeberry

Table 2 presents the results of the identification of polyphenolic compounds in chokeberry products based on the comparison of their MS and MS/MS data, retention times, UV spectra of standards and published data. Twenty-seven phenolic compounds were detected in fruits, pomace and juice from crushed and uncrushed chokeberry fruits.

The first group of phenolic compounds was related to the anthocyanin family (Peaks 1, 3, 5, 7, 10, 11, 13). The anthocyanins identified in chokeberry for the first time were: cyanidin-3,5-hexoside-(epi)catechin with [M + H]^+^ at *m*/*z* 737 and fragmentation at *m*/*z* 575, 423 and 287; cyanidin-3-pentoside-(epi)catechin with [M + H]^+^ at *m*/*z* 707 and MS/MS fragmented *m*/*z* 557, 329 and 287; and cyanidin-3-hexoside-(epi)cat-(epi)cat with the [M + H]^+^ molecular ion at *m*/*z* 1025, showing typical fragments at *m*/*z* 575, 409 and 287. These compounds were identified by the molecular ion [M − H]^+^, comparing their MS profiles with the fragmentation pathways observed, as well as the UV spectra and the retention time in pomegranate [30]. Moreover, the following were detected: cyanidin-3-*O*-galactoside and cyanidin-3-*O*-glucoside with the molecular ion [M + H]^+^ at *m*/*z* 449 and characteristic fragments at *m*/*z* 287; cyanidin-3-*O*-arabinoside with [M + H]^+^ at *m*/*z* 419 and MS/MS fragmented *m*/*z* 287; and cyanidin-3-*O*-xyloside with [M + H]^+^ at *m*/*z* 419 and fragmentation at *m*/*z* 287. Results regarding the presence of the last four anthocyanins are consistent with published data [5,29,31,32,33].

Two types of flavonol derivatives (Peaks 17–23 and 25–28) with a fragment at *m*/*z* 301 and 315, characteristic for quercetin and isorhamnetin derivatives, respectively, were found in the chokeberry products (Table 2). Quercetin derivatives are mainly flavonols found in chokeberry berries; seven quercetins as dihexoside (*m*/*z* 625), -3-*O*-vicianoside (*m*/*z* 595), 3-robinobioside and 3-*O*-rutinoside (*m*/*z* 609), -3-*O*-galactoside and -3-*O*-glucoside (*m*/*z* 463) and -*O*-deoxyhexose-deoxyhexoside (*m*/*z* 593). These results agreed with recently-published data [5,34]. Additionally, two isorhamnetin derivatives were found: -pentosylhexoside (*m*/*z* 609) and two -rhamnosyl-hexoside isomers (*m*/*z* 623). These results agreed with recently-published data [29,34]. Another group that included five derivatives of phenolic acids was identified as caffeoylquinic acid derivatives. Three of them were characterized by the same [M − H]^−^ at *m*/*z* as 353, but assigned by different compounds as: neochlorogenic acid (Rt = 2.57 min), chlorogenic acid (Rt = 3.62) [5] and cryptochlorogenic acid (Rt = 3.71), with λ_max_ = 325 nm for all. In addition, two phenolic acids were identified: 3-*O*-p-coumaroylquinic acid with [M − H]^−^ at *m*/*z* 337 and di-caffeic quinic acid with [M − H]^−^ at *m*/*z* 515, MS/MS fragments at *m*/*z* 353 and 191. Chromatography standards of neochlorogenic, chlorogenic, cryptochlorogenic and 3,5-dicaffeoylquinic acid were used to confirm the identity of these compounds. These compounds were previously found by Slimestad et al. [34] and Lee et al. [33].

The next group of phenolic compounds identified in chokeberry products by the LC-PDA-ESI-MS/MS analysis belonged to the flavan-3-ol family and included monomers and dimers. (+)-Catechin and (−)-epicatechin had an [M − H]^−^ at *m*/*z* 289. Chromatography with a standard was used to confirm their identity. Besides these compounds, one procyanidin dimer with *m*/*z* 577 [5] and one flavanone, i.e., eriodictyol-glucuronide with [M − H]^−^ at *m*/*z* 463 and an MS/MS fragment at *m*/*z* 287, were found. The latter compound is largely responsible for the bitter taste of chokeberry. It was detected for the first time in chokeberry fruits by Slimestad et al. [34].

In addition, the sum of polymeric proanthocyanidins was determined in the chokeberry products using the phloroglucinol method by UPLC-MS/MS. This method provides more detailed information on the proanthocyanidin fraction of these berries and products, especially when these compounds are difficult to detect in the UPLC-PDA analysis.

### 2.3. Comparison of Phenolic Compounds Detected in Black Chokeberry Products

**Figure 1:**
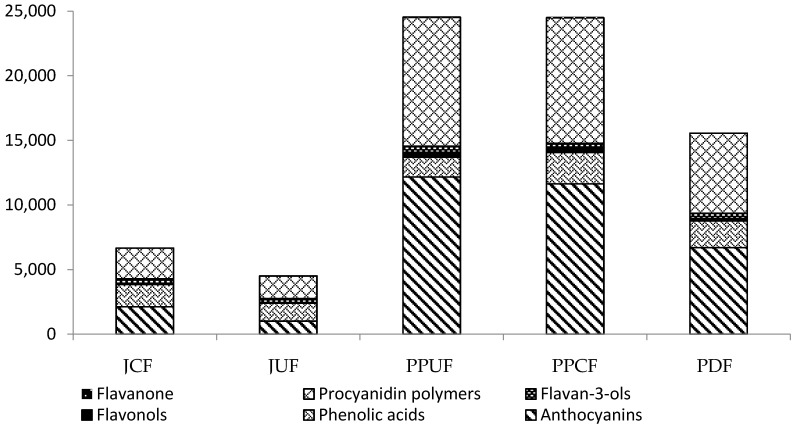
Polyphenol group content in the black chokeberry products (mg/100 g dm). Abbreviations: JCF, juice of crushed fruits before pressing; JUF, juice of uncrushed fruits before pressing; PPUF, dry powder from pomace of uncrushed fruits; PPCF, dry powder from pomace of crushed fruits; PDF, dry powder of whole fruits.

The results of the determinations of the contents of polyphenolic compounds (PC) in the analyzed samples are presented in Table 3 and in Figure 1. Their content in juices was significantly affected by the procedure of pulp preparation for pressing. In JCF, the content of polyphenols was higher by over 32% than in JUF. In the case of dried powders, the highest content of PC was obtained in the PPUF (24.45 g/100 g dm) and PPCF (15.61 g/100 g dm), and these values were significantly lower compared to the PDF (24.72 g/100 g dm). The main PC in the analyzed chokeberry products were: anthocyanins > procyanidin polymers >> phenolic acids ≥ flavonols > flavan-3-ols > flavanone. In an earlier study, Oszmiański et al. [5] determined the total phenolic compounds in pomace from black chokeberry at 10.58 g/100 g dm, which was 2.3-times lower compared to the results obtained in this study for pomace and 1.5-times lower compared to whole dried fruits. These differences may be due to various chokeberry cultivation conditions and location and to various methods of material pretreatment for the preparation of products. In a study by Horszwald et al. [1], who analyzed chokeberry powders made by different drying processes, the content of polyphenolics in freeze-dried chokeberry fruits was 27.63 g/100 g dm. This value differed insignificantly compared to the polyphenolic content determined in the powders made of pomace in our study, but was ca. two-times higher compared to PDF.

The main PC identified in chokeberry powders were anthocyanins, which constituted ~50% of total polyphenols (Table 3). The total content of anthocyanins in the analyzed samples ranged from 6.68 in PDF to 12.16 g/100 g dm in PPUF. These results confirm that anthocyanins occur mainly in skin, wherein their concentration is the highest compared to the other morphological parts of fruits. Migration of anthocyanins from fruit skin to juice is determined, most of all, by skin damage. The appropriate crushing before pressing causes damage to cell walls and thus facilitates the migration of fruit components to the pressed juice. Pectolytic enzymes are often applied as they cause degradation of cell walls, which enables the degradation of the tissue structure, forming pectins. This process allows for an additional increase in pressing yield and, consequently, in the anthocyanin content of juice [35]. These processes have a great effect on the concentration of anthocyanins in the end product [36]. In the analyzed juices, anthocyanins were also the group of compounds with the highest concentrations, which ranged from 1.01 JUF to 2.12 g/100 g dm in JCF. The method of pulp preparation resulted in over two-fold differences in the anthocyanin content of juices. The content of anthocyanins in dried powders made of juice with the freeze-drying method determined by Horszwald et al. [1] was 22.81 g/100 g dm, was 1.9-times higher compared to dry pomace and 3.4-times higher compared to whole dried fruits in our study. Differences in the contents of anthocyanins may result mostly from the juice production process. Procyanidin polymers (PP) were the second group of compounds in terms of the content in the analyzed products and represented ~40% of the total polyphenolic contents. They constitute an important group of health-promoting compounds and are responsible for the pungent taste of chokeberry. Procyanidin oligomers exhibit high affinity to proteins, thereby causing their denaturation. Such an effect is perceptible during the consumption of chokeberry fruits. However, PP are interesting due to their strong anti-inflammatory effects and beneficial impact on health, including antitumor and antiproliferative activities [17]. 

The JUF contained over 1.6-times less PP than JCF (Table 3), i.e., 1.47 and 2.37 g/100 g dm, respectively. The change in the mode of pulp preparation for pressing allowed the content of pungent and bitter PP to be reduced, which improving the organoleptic characteristics of the juice. Like anthocyanins, these compounds occur mainly in fruit skin: therefore, if whole fruits are crushed, they remain in the pomace.

In dry powders that may be applied as additives to nutraceuticals with health-promoting properties wherein a high content of PP is highly desirable, a considerably higher content of these compounds was obtained in dried pomace than in dried fruits. In our earlier investigations [5], the content of PP in pomace was 8.19 g/100 g dm, which was 1.2-times lower compared to dried pomace and 1.3-times higher compared to dried fruits in the present study. An important compound identified in chokeberry products was eriodictyol-glucuronide, belonging to the group of flavanones, which represented ~0.01% of the total polyphenol contents. It is largely responsible for the bitter taste of chokeberry fruits. Its content in juices was significantly affected by the method of their preparation, i.e., JUF or JCF. The crushing of fruits before pressing caused a 1.5-fold increase in its content in the JUF. As in the case of PP, reduction of the content of eriodictyol-glucuronide improved the taste of chokeberry juice [6].

Another group of compounds identified in chokeberry included phenolic acids (PA), which constituted ~7.2% of total phenolics (Table 2 and Table 3). Chlorogenic acid was found to predominate, with its mean contents being 0.94 in powders and 0.56 g/100 g dm in juices. In turn, the lowest contents were determined for di-caffeic quinic acid, i.e., 0.001 in juices and 0.004 g/100 g dm in powders. PA occur in various morphological parts of fruits, in both soft pulp and hard skin. Therefore, their contents were insignificantly affected by the method of powder and juice preparation (Table 2 and Table 3). The only difference, compared to the contents of anthocyanins and procyanidin polymers in the analyzed samples, was a higher content of PA in the PPUF than PPCF, i.e., 2.23 and 1.48 g/100 g dm, respectively. This may be due to a higher degree of PA oxidation during fruit crushing before pressing, as it is common knowledge that PA are good substrates of the enzyme phenoloxidase and that they are readily oxidized in enzymatic reactions.

Flavonols constituted another group of PC of chokeberry with the content representing ~1.5% of total polyphenols. Wang et al. [37] reported in their study that, compared to anthocyanins, flavonols displayed higher antioxidative activity measured with the ORAC test. Like anthocyanins, flavonols occur mainly in fruit skin; therefore, their concentration in the JCF was over 2.4-times higher than in the juice made of whole fruits. In addition, their content was ca. two-fold higher in powder made of pomace than in PDF.

Another group of compounds occurring in black chokeberry and constituting ~1.3% of the total polyphenolics was flavan-3-ols (Table 2 and Table 3). The JCF were characterized by a 1.2-times higher concentration of these compounds compared to the JUF. As in the case of PA, the PPUF contained more flavan-3-ols compared to PPCF, i.e., 0.44 and 0.31 g/100g·dm, respectively. 

### 2.4. Antioxidant Activity of Powder and Juice with Black Chokeberry

The results of the ABTS test assaying the capability to reduce free radicals and of the FRAP test assaying the capability to reduce ferric ions are presented in Table 4. The analysis of the antioxidative activity (AA) demonstrated differences in the ABTS and FRAP values between JCF and JUF and between PDF, PPUF and PPCF (Table 4). The JCF were characterized by 1.6- and 2.1-times higher ABTS and FRAP values compared to JUF. This was due to the differences in the contents of polyphenolic compounds. The results of the ABTS test conducted for JCF (32.73 mmol Trolox/100 g dm) are comparable to the findings of previous studies carried out with chokeberry juices (31.41 mmol Trolox/100 g dm) [5].

Comparison of the AA of powders revealed significantly higher values in the samples of dried chokeberry fruits than in those of dried pomace. The results of the ABTS method were 81.66 and 81.63 mmol Trolox 100 g·dm for PDF and PPUF, respectively, whereas the results of the FRAP method were 53.78 and 52.22 mmol Trolox/100 g dm, respectively. A considerably lower capability to reduce free ABTS radicals and of ferric ions was found for the PPCF, i.e., 59.94 and 32.61 mmol Trolox/100 g dm, respectively.

The AA determined with the ABTS test by Oszmiański et al. [5] in pomace was 77.96 mmol Trolox/100 g dm and was slightly higher than that assayed in the reported study in pomace samples: 81.63 mmol Trolox/100 g dm. According to Horszwald et al. [1], the capability to reduce free radicals determined with the ABTS method in powders made of freeze-dried chokeberry juice was 180.45 mmol Trolox/100 g dm and was two- and three-times higher compared to those assayed in dried pomace and fruits. In turn, the capability to reduce ferric ions analyzed with the FRAP method in chokeberry powders was 193.69 mmol Trolox/100 g dm and was ca. 3.5-times higher than in dried pomace and fruits. The difference was due to the use of various raw material, because in the work by Horszwald et al. [1], the dried materials were made of juice, whereas in the present study, they derived from chokeberry fruits. In addition, the results could be affected by differences in the technology of juice production.

### 2.5. Effect of Chokeberry Pre-Treatment on the Content of Phenolic Compounds and Antioxidant Activity in Final Products

The results support the feasibility of modifying the production process of chokeberry juices and powders. The JUF enabled positive results to be achieved, as the juice was characterized by a lower content of compounds responsible for its pungent and bitter taste. In turn, pomace turned out to be a better raw material than fruits for the production of chokeberry powders, because it is easier to dry, requires less time and energy to evaporate water and contains more bioactive substances.

In order to depict differences in the contents of the analyzed components depending on the method of fruit pre-treatment for the production of chokeberry juices and powders, their percentage contents were calculated. The degree of fruit disintegration affected the juice pressing yield. The yield of JCF was 67%, which was significantly higher compared to the yield of JUF (47%). Large differences were found in the contents of sugars and polyphenols between juices obtained in particular variants. A greater amount of sugar (56%) migrated to JCF than during JUF (44%). The pressing of crushed fruits results in a higher content of polyphenols (70%) in juices. In the case of pressing the whole fruits, only 30% of these compounds were found in juice. This way of juice production contributed to a significantly higher content of bioactive compounds remaining in the pomace. Owing to this, the production of chokeberry PPUF material has two advantages. These juices have lower contents of PC and, therefore, are characterized by a milder taste. These compounds are present mainly in fruit skin and remain in pomace after pressing. This makes the pomace a valuable material for the production of powders with a high biological activity [34]. In addition, during drying, the highest volume of water was evaporated from whole chokeberry fruits, i.e., 81%, whereas in pomace from whole fruits, it was 70%, and in pomace from crushed fruits, it was 56%. The lower the water content in the raw materials was, the higher was the yield of the drying process, i.e., 18.8%, 30.1% and 43.5%, respectively. The drying process for chokeberry pomace is easier and faster, and the pomace required the evaporation of a significantly smaller volume of water because ca. 50% of water was eliminated during drying.

The berry pre-treatment step significantly (*p* < 0.05) affected the content of PC and AA in the final product obtained from PPUF and the contents of TPC and AA in the products from PPCF. A similar effect on PC was observed in blueberry products [38]. The effects of berry pre-treatment on AA were previously described by other authors [39]. Moreover, pre-treatment of berries before pressing significantly (*p* < 0.05) affected the contents of PC and AA in the final juice obtained from crushed fruit compared to juice from uncrushed fruit.

The pre-treatment of berries significantly (*p* < 0.05) affected final PC and AA in powders and juice. The powders obtained from uncrushed fruit showed significantly more (1.4-times) AA and 1.6-times more PC than crushed berries. Positive correlations were found for TPC content (total phenolics, anthocyanins, flavan-3-ols, flavanols and PA) and AA (the ABTS and FRAP methods) (Table 5). These correlations showed that the AA of chokeberry products depends mainly on the content of PC.

## 3. Materials and Methods

### 3.1. Reagents and Standards

Acetonitrile, formic acid, methanol, ABTS (2,2′-azinobis-(3-ethylbenzothiazoline-6-sulphonic acid), Trolox (6-hydroxy-2,5,7,8-tetramethylchroman-2-carboxylic acid), TPTZ (2,4,6-Tri(2-pyridyl)-s-triazine), acetic acid phloroglucinol and eriodictyol were purchased from Sigma-Aldrich (Steinheim, Germany). (−)-Epicatechin, (+)-catechin, isoquercitrin, quercetin 3-*O*-glucoside, quercetin 3-*O*-galactoside, isorhamnetin 3-*O*-glucoside, chlorogenic acid, neochlorogenic acid, cryptochlorogenic acid, di-caffeic quinic acid, procyanidin B2, *p*-coumaric acid, caffeic acid, cyanidin-3-*O*-galactoside and cyanidin-3-*O*-glucoside were purchased from Extrasynthese (Lyon, France).

### 3.2. Plant Materials

Fruits of chokeberry cv. Galicjanka (~15 kg) were obtained from a horticultural farm in Trzebnica, near Wrocław (Poland). The raw material was collected at the optimum ripening stage recommended for consumption. The juice was pressed from crushed (JCF) and uncrushed fruits (JUF) on a Zodiak laboratory press. The chokeberry fruit was crushed or not crushed, and then, these two variants of materials were pressed on a hydraulic press (SSRE, Warsaw, Poland) (at a piston thrust of 15 tons of pressure of 2 min). The whole fruit (as a control) and pomace from crushed and uncrushed fruit were dried using an Alpha 1–4 LSC freeze dryer (Christ, Osterode, Germany). During the freeze drying, the pressure was reduced to 0.960 kPa. The temperature in the drying chamber was −60 °C, while the temperature of shelves reached 26 °C. In the next step, the materials were ground using a closed laboratory mill (IKA A.11; Christ, Osterode, Germany) to avoid hydration, and the powder was passed through a strainer (1 mm). As a result, three powder products were obtained from dried fruits (PDF), pomace of crushed (PPCF) and uncrushed fruits (PPUF). The dry matter was determined in fruit, pomaces and juice from whole and crushed fruits of chokeberry by Oszmiański et al. [40]. Results are reported as the arithmetic mean of three independent repetitions, taking into account the standard deviation (SD).

### 3.3. Extraction Procedure

The powder samples of fruits and pomace (2 g) were extracted with 50 mL of methanol acidified with 2.0% formic acid. The extraction was performed twice by incubation for 20 min under sonication (Sonic 6D, Polsonic, Warsaw, Poland) and with occasional shaking. Next, the slurry was centrifuged at 19,000× *g* for 10 min, and the supernatant was filtered through a Hydrophilic PTFE 0.20-m membrane (Millex Samplicity Filter, Merck, Darmstadt, Germany) and used for analysis. The content of polyphenols in individual extracts was determined by means of the ultra-performance liquid chromatography-photodiode array detector-mass spectrometry (UPLC-PDA-MS, Manchester, UK) method. All extractions were carried out in triplicate. The samples were determined according to the method described by Oszmiański et al. [40].

### 3.4. Qualitative and Quantitative Assessment of Polyphenols

Identification and quantification of polyphenol (anthocyanins, flavan-3-ols, flavonols, flavanone and phenolic acids) of black chokeberry extracts was carried out using an ACQUITY Ultra Performance LC system equipped with a photodiode array detector with a binary solvent manager (Waters Corporation, Milford, MA, USA) series with a mass detector G2 Q/TOF micro mass spectrometer (Waters, Manchester, UK) equipped with an electrospray ionization (ESI) source operating in negative and positive modes. Separations of individual polyphenols were carried out using a UPLC BEH C18 column (1.7 m, 2.1 × 100 mm, Waters Corporation, Milford, MA; USA) at 30 °C. The samples (10 µL) were injected, and the elution was completed in 15 min with a sequence of linear gradients and isocratic flow rates of 0.45 mL·min^−1^. The mobile phase consisted of Solvent A (2.0% formic acid, *v*/*v*) and Solvent B (100% acetonitrile). The program began with isocratic elution with 99% Solvent A (0–1 min), and then a linear gradient was used until 12 min, reducing Solvent A to 0%; from 12.5–13.5 min, the gradient returned to the initial composition (99% A), and then, it was held constant to re-equilibrate the column. The analysis was carried out using full-scan, data-dependent MS scanning from *m*/*z* 100 to 1500. Leucine enkephalin was used as the reference compound at a concentration of 500 pg/L, at a flow rate of 2 L/min, and the [M − H]^−^ ion at 554.2615 Da was detected. The [M − H]^−^ ion was detected during a 15-min analysis performed within ESI-MS accurate mass experiments, which were permanently introduced via the Lock-Spray channel using a Hamilton pump. The lock mass correction was ±1.000 for the mass window. The mass spectrometer was operated in negative and positive ion mode, set to the base peak intensity (BPI) chromatograms and scaled to 12,400 counts per second (cps) (100%). The optimized MS conditions were as follows: capillary voltage of 2500 V, cone voltage of 30 V, source temperature of 100 °C, desolvation temperature of 300 °C and desolvation gas (nitrogen) flow rate of 300 L/h. Collision-induced fragmentation experiments were performed using argon as the collision gas, with voltage ramping cycles from 0.3–2 V. Characterization of the single components was carried out via the retention time and the accurate molecular masses. Each compound was optimized to its estimated molecular mass [M − H]^−^/[M+H]^+^ in the negative and positive mode before and after fragmentation. The data obtained from UPLC-MS were subsequently entered into the MassLynx 4.0 ChromaLynx Application Manager software. On the basis of these data, the software is able to scan different samples for the characterized substances. The runs were monitored at the wavelength of 360 nm for flavonol glycosides. The PDA spectra were measured over the wavelength range of 200–800 nm in steps of 2 nm. The retention times and spectra were compared to those of the pure standard. The calibration curves were run at 360 nm for the standard myricetin, at 320 nm for the standard of chlorogenic, caffeic, *p*-coumaric and sinapic acid, at 520 nm for the standard delphinidin 3-*O*-galactoside, peonidin 3-*O*-glucoside, cyanidin 3-*O*-galactoside and malvidin 3-*O*-glucoside and at 280 nm for the standard (−) epicatechin, (+)-catechin, procyanidins B_2_ and A, at concentrations ranging from 0.05–5 mg/mL (*R*^2^ = 0.9999). All measurements were repeated three times. The results are presented as mg per 100 g dry matter (dm) and TP as g per 100 g dm.

### 3.5. Analysis of Proanthocyanidins by Phloroglucinolysis Method

Direct phloroglucinolysis of freeze-dried samples was performed as described by Oszmiański et al. [40]. Portions (0.5 mL) of juices were precisely measured into 2-mL Eppendorf vials and freeze-dried. Fruit and pomace lyophilisates were weighed in an amount of 5 mg into 2-mL Eppendorf vials. Subsequently, 0.8 mL of the methanolic solution of phloroglucinol (75 g/L) and ascorbic acid (15 g/L) were added to samples. After addition of 0.4 mL of methanolic HCl (0.3 M), the vials were incubated for 30 min at 50 °C with continuous vortexing in a thermo shaker (TS-100, BioSan, Riga, Latvia). The reaction was terminated by placing the vials in an ice bath, drawing 0.6 mL of the reaction medium and diluting with 1.0 mL of sodium acetate buffer (0.2 M). The samples were centrifuged immediately at 20,000× *g* for 10 min at 4 °C and stored at 4 °C before reverse-phase HPLC (RP-HPLC) analysis. All incubations were done in triplicate. Phloroglucinolysis products were separated on a Cadenza CD C18 (75 mm × 4.6 mm, 3 m) column (Imtakt, Kyoto, Japan). The liquid chromatograph was a Waters (Milford, MA, USA) system equipped with diode array and scanning fluorescence detectors (Waters 474) and an autosampler (Waters 717 plus). Solvent A (25 mL aqueous acetic acid and 975 mL water) and Solvent B (acetonitrile) were used in the following gradients: initial, 5% B; 0–15 min to 10% B linear; 15–25 min to 60% B linear. This was followed by washing and reconditioning of the column. Other parameters were as follows: a flow rate of 1 mL/min, an oven temperature of 15 °C and a volume of filtrate injected onto the HPLC system of 20 L. The compounds for which reference standards were available were identified in chromatograms according to their retention times and UV-visible spectra. The fluorescence detection was recorded at excitation wavelength of 278 nm and emission wavelength of 360 nm. The calibration curves, which were based on peak area, were established using (+)-catechin, (−)-epicatechin, (+)-catechins and (−)-epicatechin-phloroglucinol adduct standards. The average degree of polymerization was measured by calculating the molar ratio of all the flavan-3-ol units (phloroglucinol adducts + terminal units) to (−)-epicatechin and (+)-catechin, which correspond to terminal units. Quantification of the (+)-catechin, (−)-epicatechin, (+)-catechin and (−)-epicatechin-phloroglucinol adducts was achieved by using the calibration curves of the corresponding standards (Extrasynthese). All measurements were repeated three times. The results are presented as mg per 100 g dm.

### 3.6. Ferric-Reducing/Antioxidant Power Assay

The solvent for analysis was prepared and described previously by Lachowicz et al. [41]. The total antioxidant potential of samples was determined using a ferric-reducing ability of plasma (FRAP) assay [42] as a measure of antioxidant power and juice. The assay was based on the reducing power of a compound (antioxidant). A potential antioxidant will reduce the ferric ion (Fe^3+^) to the ferrous ion (Fe^2+^); the latter forms a blue complex (Fe^2+^/TPTZ), which increases the absorption at 593 nm. Briefly, the FRAP reagent was prepared by mixing acetate buffer (300 mM, pH 3.6), a solution of 10 mM TPTZ in 40 mM HCl and 20 mM FeCl_3_ at 10:1:1 (*v*/*v*/*v*). The reagent (3 mL) and sample solutions (1 mL) were added to each well and mixed thoroughly. The absorbance was taken at 593 nm after 10 min. A standard curve was plotted using different concentrations of Trolox. All solutions were used on the day they were prepared. The results were corrected for dilution and expressed in mmol of Trolox equivalent per 100 g of sample dry matter. All determinations were performed in triplicate using a Shimadzu UV-2401 PC spectrophotometer (Kyoto, Japan).

### 3.7. Free Radical Scavenging Ability Determination Using a Stable ABTS Radical Cation

The solvent for analysis was prepared and described previously by Lachowicz et al. [41]. The free radical scavenging activity was determined by the ABTS radical cation decolorization assay, described by Re et al. [43]. ABTS was dissolved in water to a 7 mM concentration. The ABTS radical cation (ABTS+) was produced by reacting ABTS stock solution with 2.45 mM potassium persulfate (final concentration) and kept in the dark at room temperature for 12–16 h before use. The radical was stable in this form for more than two days when stored in the dark at room temperature. The samples containing the ABTS+ solution were diluted with redistilled water to an absorbance of 0.700 (±0.02) at 734 nm and equilibrated at 30 °C. After the addition of 3.0 mL of diluted ABTS+ solution to 30 L of polyphenolic extracts, the absorbance was read exactly 6 min after initial mixing. The results were corrected for dilution and expressed in mmol of Trolox equivalent per 100 g of sample dm. All determinations were performed in triplicate using a Shimadzu UV-2401 PC spectrophotometer (Kyoto, Japan).

### 3.8. Analysis of Sugar by the HPLC-ELSD Method

The samples of chokeberry fruits (4–5g) were diluted with redistilled water (50 mL). The extraction was performed by incubation for 15 min under sonication (Sonic 6D, Polsonic, Warsaw, Poland) and with occasional shaking and then incubation at 90 °C for 30 min. Next, the slurry was centrifuged at 19,000× *g* for 10 min, and the supernatant was filtered through Sep-Pak C-18 Cartridges (Waters Millipore) and through a Hydrophilic PTFE 0.20-mm membrane (Millex Samplicity Filter, Merck) and used for analysis. All extractions were carried out in triplicate. Chromatographic analysis was carried out with a Merck-Hitachi L-7455 liquid chromatograph with an evaporative light scattering detector (ELSD; Polymer Laboratories PL-ELS 1000) and the quaternary pump L-7100 equipped with the D-7000 HSM Multisolvent Delivery System (Merck-Hitachi, Tokyo, Japan) and an L-7200 autosampler. The separation was performed on a Prevail™ Carbohydrate ES HPLC Column-W 250 4.6 mm, 5 mm (Imtakt, Kyoto, Japan) column. Oven temperature was set to 3 0°C. The mobile phase was used with an acetonitrile water mixture (75:25) for isocratic elution; the flow rate was 1 mL/min and injection volume of 10 mL. The ELS detector was optimized for the analyses, and the following parameters were used: 80 °C for an evaporative temperature, 80 °C for the nebulizer and 1.2 mL/min for the nitrogen gas flow. Calibration curves (R^2^ ¼ 0.9998) were created for glucose, fructose and saccharine. Sample concentrations were 1, 2, 3 and 5 mg/mL, and each concentration level was injected (10 mL) in triplicate. All measurements were repeated three times. The results are presented as g per 100 g dm. 

### 3.9. Statistical Analysis

Results are presented as the mean ± standard deviation of three independent determinations. All statistical analyses were performed with Statistica Version 12.5 (StatSoft, Krakow, Poland). One-way analysis of variance (ANOVA) by Duncan’s test was used to compare the mean values.

## 4. Conclusions

The proposed modification of the process of chokeberry juice production from uncrushed rather than from crushed fruits had a positive effect on taste improvement. The achieved lower content of polyphenolic compounds, particularly proanthocyanidin polymers and eriodictyol-glucuronide, probably contributed to the attenuation of the pungent and bitter taste of the juice produced. Furthermore, chokeberry pomace turned out to be a richer source of bioactive polyphenols compared to dried fruits. The best results were achieved for powders from the pomace of uncrushed fruits. Due to lower water content, lyophilization and crushing of pomace were easier than in the case of fruit. Similar relationships were observed for antioxidant activity, which was the highest in the dried pomace of uncrushed berry. Pomace may be dried more easily and is less hygroscopic, because it contains less sugar. It may be used for the production of functional foods in the form of pills or powder or as a food additive aimed at improving the biological value of food.

## Figures and Tables

**Table 1:** Chemical composition of samples from black chokeberry.

Chemical Compounds	JUF ^1^	JCF ^1^	PDF ^1^	PPUF ^1^	PPCF ^1^
Dry substance (%)	16.87 ± 0.07 d ^2^	15.46±0.07 e	96.86 ± 0.05 c	97.63 ± 0.07 b	98.30 ± 0.08 a
Fructose (g/100 g dm)	19.18 ± 0.03 a	19.04 ± 0.03 b	10.43 ± 0.02 c	6.73 ± 0.02 d	6.64 ± 0.03 e
Sorbitol (g/100 g dm)	41.99 ± 0.03 a	39.29 ± 0.04 b	21.08 ± 0.04 c	12.55 ± 0.03 e	13.33 ± 0.03 d
Glucose (g/100 g dm)	26.15 ± 0.08 a	26.91 ± 0.03 a	14.64 ± 0.06 b	12.55 ± 0.07 c	5.08 ± 0.04 d
Total sugar	87.31 ± 0.03 a	85.24 ± 0.06 b	46.15 ± 0.08 c	31.83 ± 0.05 d	25.05 ± 0.06 e

^1^ JUF, juice from uncrushed fruit; JCF, juice from crushed fruit; PDF, powder from dried fruit; PPUF, powder from pomace of uncrushed fruit; PPCF, powder from pomace of crushed fruit; ^2^ a–e: means ± SD followed by different letters within the same line represent significant differences (*p* < 0.05); data are the averages of triplicates.

**Table 2:** Groups of phenolic compounds identified by LC-PDA-ESI-MS/MS in black chokeberry products.

Compounds ^1^	Rt (min)	λ_max_ (nm)	[H − M]^−^ (*m*/*z*) ^2^	MS/MS Fragments (*m*/*z*) ^2^
Cyanidin-3-hexoside-(epi)catechin	2.54	520	737+	575/423/287
Neochlorogenic acid	2.57	323	353	191
Cyanidin-3-pentoside-(epi)catechin	2.98	520	707+	557/329/287
(+)-Catechin	3.04	280	289	
Cyanidin-3-hexoside-(epi)cat-(epi)cat	3.15	520	1025+	575/409/287
3-*O*-*p*-Coumaroylquinic acid	3.30	310	337	191
Cyanidin-3-*O*-galactoside	3.50	516	449+	287
Chlorogenic acid	3.62	323	353	191
Cryptochlorogenic acid	3.71	323	353	191
Cyanidin-3-*O*-glucoside	3.79	517	449+	287
Cyanidin-3-*O*-arabinoside	4.02	515	419+	287+
Procyanidin B2	4.20	280	577	289
Cyanidin-3-*O*-xyloside	4.65	515	419+	287+
(−)-Epicatechin	4.88	280	289	
Quercetin-dihexoside	5.23	352	625	445/301
Quercetin-dihexoside	5.29	352	625	445/301
Quercetin-3-*O*-vicianoside	5.50	353	595	432/301
Quercetin-3-robinobioside	5.84	353	609	463/301
Quercetin-3-*O*-rutinoside	6.02	353	609	463/301
Quercetin-3-*O*-galactoside	6.09	352	463	301
Quercetin-3-*O*-glucoside	6.22	352	463	301
Eriodictyol-glucuronide	6.28	280	463	287
Isorhamnetin pentosylhexoside	6.41	352	609	315
Quercetin-*O*-deoxyhexose-deoxyhexoside	6.76	352	593	433/301
Isorhamnetin rhamnosyl hexoside isomer	6.72	352	623	463/315
Isorhamnetin rhamnosyl hexoside isomer	6.89	352	623	421/315
Di-caffeic quinic acid	6.94	323	515	353/191

^1^ Identification confirmed by commercial standards; ^2^ experimental data.

**Table 3:** Polyphenol contents in black chokeberry products (mg/100 g dm) ^1^.

Compounds	JUF	JCF	PDF	PPUF	PPCF
Cyanidin-3,5-hexoside-(epi)catechin	9.16 ± 0.07 c	9.87 ± 0.08 b ^2^	12.04 ± 0.06 e	20.43 ± 0.09 a	14.33 ± 0.07 d
Neochlorogenic acid	891.56 ± 4.89 c	1048.49 ± 4.84 b	728.81 ± 3.53 d	1174.35 ± 4.38 a	1161.53 ± 3.71 b
Cyanidin-3-pentoside-(epi)catechin	3.95 ± 0.03 e	4.24 ± 0.04 c	5.76 ± 0.03 d	10.30 ± 0.05 a	7.26 ± 0.04 b
(+)-Catechin	87.66 ± 0.68 e	107.18 ±0.90 d	122.70 ± 0.59 a	180.27 ± 0.83 b	142.81 ± 0.70 c
Cyanidin-3-hexoside-(epi)cat-(epi)cat	6.77 ± 0.05 e	7.74 ± 0.07 d	10.98 ± 0.05 a	20.23 ± 0.09 b	13.61 ± 0.07 c
3-*O*-*p*-Coumaroylquinic acid	8.31 ± 0.06 e	9.32 ± 0.08 d	6.81 ± 0.03 a	12.70 ± 0.06 b	10.96 ± 0.05 c
Cyanidin-3-*O*-galactoside	702.15 ± 4.43 e	1451.55 ± 4.24 d	8286.4 ± 4.83 b	7961.70 ±4.48 a	4521.34 ± 2.22 c
Chlorogenic acid	470.51 ± 3.64 e	642.74 ± 4.42 d	769.25 ± 3.73 a	1 192.69 ±3.46 b	848.17 ± 4.17 c
Cryptochlorogenic acid	28.21 ± 0.22 b	32.66 ± 0.28 a	19.91 ± 0.10 e	53.60 ± 0.25 d	41.57 ± 0.20 c
Cyanidin-3-*O*-glucoside	19.71 ± 0.15 b	39.99 ± 0.34 a	225.80 ± 1.09 e	220.06 ± 1.01 c	125.91 ± 0.62 d
Cyanidin-3-*O*-arabinoside	248.72 ± 1.92 e	554.90 ± 2.68 b	3328.79 ± 2.21 d	3116.02 ±2.28 a	1835.62 ± 9.02 c
Procyanidin B2	21.90 ± 0.17 c	28.19 ± 0.24 a	24.86 ± 0.12 e	42.13 ± 0.19 b	36.40 ± 0.18 d
Cyanidin-3-*O*-xyloside	17.32 ± 0.13 e	48.35 ± 0.41 c	294.14 ± 1.42 b	275.41 ± 1.26 a	166.86 ± 0.82 d
(–)-Epicatechin	213.58 ± 1.65 e	235.28 ± 1.98 d	174.53 ± 0.85 a	260.13 ± 1.19 b	236.19 ± 1.16 c
Quercetin-dihexoside	1.89 ± 0.01 e	4.15 ± 0.04 d	30.99 ± 0.15 a	30.35 ± 0.14 b	15.19 ± 0.07 c
Quercetin-dihexoside	1.00 ± 0.01 e	2.21 ± 0.02 d	12.59 ± 0.06 a	12.80 ± 0.06 b	6.80 ± 0.03 c
Quercetin-3-*O*-vicianoside	1.95 ± 0.02 e	5.50 ± 0.05 d	45.32 ± 0.22 b	43.20 ± 0.20 a	20.41 ± 0.10 c
Quercetin-3-robinobioside	4.94 ± 0.04 e	10.75 ± 0.09 d	47.45 ± 0.23 a	50.52 ± 0.23 b	25.60 ± 0.13 c
Quercetin-3-*O*-rutinoside	4.29 ± 0.03 e	8.98 ± 0.08 d	44.31 ± 0.21 a	43.68 ± 0.20 b	22.74 ± 0.11 c
Quercetin-3-*O*-galactoside	6.77 ± 0.05 e	16.46 ± 0.14 d	104.11 ± 0.50 b	102.43 ± 0.47 a	48.97 ± 0.24 c
Quercetin-3-*O*-glucoside	7.08 ± 0.05 e	13.54 ± 0.11 c	63.27 ± 0.31 b	67.14 ± 0.31 a	32.75 ± 0.16 d
Eriodictyol-glucuronide	19.24 ± 0.15 e	28.97 ± 0.24 d	81.36 ± 0.39 a	84.40 ± 0.39 b	57.61 ± 0.28 c
Isorhamnetin pentosylhexoside	0.30 ± 0.00 d	0.81 ± 0.01 c	4.33 ± 0.02 b	12.20 ± 0.06 a	0.81 ± 0.00 c
Quercetin-*O*-deoxyhexose-deoxyhexoside	0.19 ± 0.00 e	0.58 ± 0.00 d	1.96 ± 0.01 a	3.04 ± 0.01 b	1.31 ± 0.01 c
Isorhamnetin rhamnosylhexosideisomer	0.49 ± 0.00 e	1.16 ± 0.01 d	12.05 ± 0.06 a	9.16 ± 0.04 b	5.18 ± 0.03 c
Isorhamnetin rhamnosylhexosideisomer	0.25 ± 0.00 e	0.69 ± 0.01 d	6.36 ± 0.03 a	5.11 ± 0.02 b	2.77 ± 0.01 c
Di-caffeic quinic acid	1.00 ± 0.01 e	1.33 ± 0.01 d	4.88 ± 0.02 b	5.35 ± 0.02 a	3.09 ± 0.02 c
Procyanidin polymers	1472.27 ± 4.21 e	2371.07 ± 4.13 d	9977.84 ±3.78 a	9714.57 ± 2.45 b	6201.73 ± 1.76 c
TP ^3^	4521.18 b	6686.69 a	24723.67 c	24447.77 d	15607.48 e
TP (g/100 g)	4.52 b	6.69 a	24.72 c	24.45 d	15.61 e

^1^ Values are the means ± standard deviation. *n* = 3; ^2^ a–e: means ± SD followed by different letters within the same line represent significant differences (*p* < 0.05); ^3^ total phenolic compounds.

**Table 4:** Antioxidant capacities of juice and dry pomace prepared from uncrushed and crushed black chokeberry fruits (mmol Trolox/100 g dm) ^1^.

Compounds	JUF	JCF	PDF	PPUF	PPCF
ABTS ^3^	20.11 ± 0.2 d ^2^	32.73 ± 0.1 c	81.66 ± 0.2 a	81.63 ± 0.2 a	59.94 ± 0.1 b
FRAP ^4^	9.81 ± 0.2 e	20.20 ± 0.0 d	53.78 ± 0.1 a	52.22 ± 0.2 b	32.61 ± 0.2 c

^1^ Values are the means ± standard deviation. *n* = 3; ^2^ a–e: means ± SD followed by different letters within the same line represent significant differences (*p* < 0.05); ^3^ ABTS, 2,2′-azinobis(3-ethylbenzothiazoline-6-sulphonic acid; ^4^ FRAP, ferric reducing antioxidant power.

**Table 5:** Correlation matrix between polyphenolic compounds and the key antioxidant activity method of chokeberry and its products.

Polyphenolic Compounds	ABTS	FRAP
Anthocyanins	0.797	0.902
Flavan-3-ols	0.790	0.752
Flavonols	0.782	0.884
Phenolic acid	0.716	0.666
Flavanone	0.804	0.908
TP	0.890	0.876
